# Tranexamic acid in radical cystectomy: a systematic review and meta-analysis of efficacy and safety

**DOI:** 10.1007/s00345-025-05939-0

**Published:** 2025-09-18

**Authors:** Maurin Helen Mangold, Victoria Luise Simone Wieland, Maurizio Grilli, Alexander Studier-Fischer, Caelán Max Haney-Aubert, Niklas Westhoff, Maurice Stephan Michel, Luisa Egen, Karl-Friedrich Kowalewski

**Affiliations:** 1 Department of Urology and Urosurgery, Medical Faculty Mannheim at Heidelberg University, University Medical Center Mannheim, Theodor-Kutzer-Ufer 1-3, 68167 Mannheim, Germany; 2 German Cancer Research Center (DKFZ) Heidelberg, Division of Intelligent Systems and Robotics in Urology (ISRU), Heidelberg, Germany; 3 DKFZ Hector Cancer Institute at the University Medical Center Mannheim, Mannheim, Germany; 4 Medical Library, Medical Faculty Mannheim, University of Heidelberg, Mannheim, Germany

**Keywords:** Tranexamic acid, Cystectomy, Blood transfusion, Venous thromboembolism

## Abstract

**Purpose:**

Radical cystectomy (RC) is a high-risk procedure, associated with significant bleeding and high risk of perioperative blood transfusion (PBT), which may limit clinical and oncological outcomes. Tranexamic acid (TXA), an antifibrinolytic agent, effectively reduces bleeding in various surgical settings; however, its role in RC remains understudied. This systematic review and meta-analysis evaluates the efficacy and safety of TXA in RC patients.

**Methods:**

A systematic search of CENTRAL, PubMed, Embase, and Web-of-Science was conducted, including studies published between January 2013 and January 2025. Four studies (one randomised controlled trial (RCT) and three retrospective cohort studies) met inclusion criteria. Data were pooled using random-effects meta-analysis. Risk of bias was assessed using ROBINS-I and RoB-2, and certainty of evidence was evaluated using GRADE.

**Results:**

TXA was associated with a significant reduction in overall PBT (odds ratio (OR) 0.48, 95%-confidence interval (CI) 0.27–0.82; *p* = 0.008), though the only RCT included failed to identify this trend. Estimated blood loss was numerically reduced in the TXA group, but did not reach statistical significance (mean difference − 36.8 mL; 95%-CI -78.1-4.6; *p* = 0.08). Notably, TXA did not increase the incidence of venous thromboembolism (OR 1.49, 95%-CI 0.86–2.57; *p* = 0.16). Key limitations include substantial heterogeneity between studies (I² = 78% for PBT outcome), varying TXA dosing protocols, and the predominance of retrospective studies.

**Conclusion:**

TXA may reduce PBT requirements in RC without increasing thromboembolic risk. Further research is needed to confirm these findings, refine dosing protocols, and assess efficacy in minimally invasive RC.

**Supplementary Information:**

The online version contains supplementary material available at 10.1007/s00345-025-05939-0.

## Introduction

Radical cystectomy (RC) with bilateral pelvic lymph node dissection remains the gold standard for patients with muscle-invasive bladder cancer and selected cases of high-risk non-muscle-invasive bladder cancer [1]. While RC offers the potential for durable oncological control, it is a technically demanding procedure associated with significant intraoperative blood loss and high risk of perioperative blood transfusion (PBT), with reported transfusion rates of up to 60% [2]. This high rate is underscored by the fact that up to 40% of patients undergoing RC are anaemic prior to surgery [3]. Although PBT can be life-saving, it carries inherent risks, including transfusion reactions and immunomodulatory effects, as well as significant healthcare costs [4, 5]. In addition, accumulating evidence suggests that PBT may adversely affect oncological outcomes, with several studies reporting an increased risk of cancer recurrence and cancer-specific mortality in patients receiving PBT during RC [2, 6]. In response to these concerns, considerable efforts have been made to minimise transfusion requirements in surgical oncology including restrictive transfusion protocols, careful crystalloid management, and intraoperative use of topical haemostatic agents. Among pharmacological interventions, antifibrinolytic agents - most notably tranexamic acid (TXA) - have received considerable attention. TXA, a synthetic lysine analogue, inhibits the activation of plasminogen to plasmin, thereby stabilizing fibrin clots, and reducing blood loss [7]. Its efficacy and safety have been well established in a variety of surgical settings, including orthopaedic, trauma and postpartum haemorrhage [8, 9]. Despite its widespread use in other surgical fields, the use of TXA in urological oncology, specifically during RC, has been understudied. Preliminary evidence from other urological procedures, such as transurethral resection of prostate and radical prostatectomy, suggests that TXA may effectively reduce transfusion requirements [10]. However, concerns about potential adverse effects, particularly venous thromboembolism (VTE), have restricted its wider use in cancer surgery [11, 12].

Given the clinical importance of reducing PBT and its associated risks, the aim of this systematic review and meta-analysis is to synthesise the available evidence on the efficacy and safety of TXA in patients undergoing RC. Specifically, we aim to evaluate the impact of TXA on perioperative blood loss, the need for PBT and the incidence of thromboembolic events, as well as potentially exploring the impact on oncological outcomes. By consolidating the existing literature, we aim to inform clinical practice and guide future research into the role of TXA in one of the most complex procedures in urological oncology.

## Materials and methods

This systematic review and meta-analysis offers a comparative analysis of four studies that examined the impact of TXA on the intra- and postoperative outcomes of patients undergoing RC. The review was conducted in accordance with the guidelines of the Preferred Reporting Items for Systematic Reviews and Meta-Analyses (PRISMA), the Cochrane Handbook for Systematic Reviews of Interventions, and the protocols of the Study Center of the German Society for Surgery [13, 14]. The research plan was formally documented and officially registered with PROSPERO (ID: CRD420251029152) prior to the initiation of the study.

### Search strategy

**Fig. 1:**
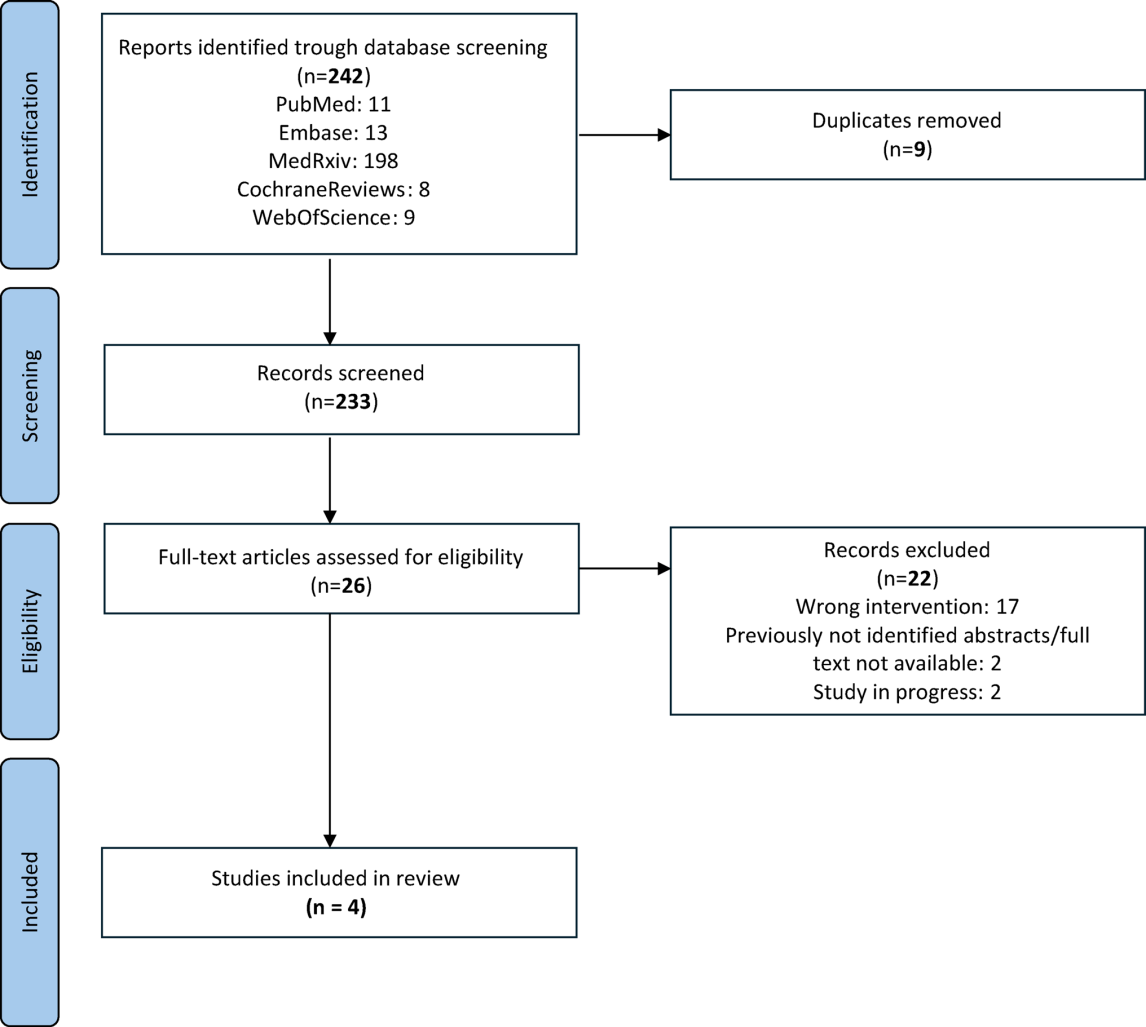
PRISMA flow diagram of the study selection process

In accordance with the recommendations set out by Goossen et al. [15], a systematic search was conducted across multiple electronic databases, namely Cochrane CENTRAL, PubMed, Embase, and Web of Science. We included studies that enrolled adult patients (> 18 years) undergoing RC in which TXA was administered. Eligible designs were randomized controlled trials (RCT), systematic reviews (SR), meta-analyses (MA), and observational studies. We excluded non-RC populations, patients < 18 years, case reports, and animal or in-vitro studies. The database search was conducted on January 30, 2025, and included studies published between January 2013 and January 2025. A search for ongoing trials was conducted using two sources: ClinicalTrials.gov and the WHO ICTRP. The search was conducted with the assistance of a librarian (M.G.) and followed the PICOS framework [16]. The full search strategy is described in detail in Fig. 1.

*The following* ***PICOS*** *criteria were used*:

**P** (patients): Patients aged > 18 years undergoing radical cystectomy.

**I** (intervention): Tranexamic Acid.

**C** (comparator): none.

**O** (outcome): not defined for search in order to increase sensitivity.

**S** (study design): RCTs, SR, MA, and observational studies.

### Data collection and analysis

Two reviewers (MHM and LE) independently assessed the titles, abstracts and full texts of the articles identified in the literature search to determine their eligibility based on aforementioned inclusion criteria. References were imported into EndNote 20 (Clarivate Analytics, London, UK) and duplicates were removed. Dichotomous outcomes were documented by recording event counts and total number of participants. Means (M) and standard deviations (SD) were recorded for continuous outcomes. In the case of missing data, the study authors were contacted or missing values (e.g., means and SDs) were imputed using standard methods from the Cochrane Handbook for Systematic Reviews of Interventions. The study characteristics were reviewed for each included study.

### Statistical analysis

- Dichotomous outcomes were expressed as odds ratios (OR) with 95% confidence intervals (CI), calculated using the Mantel-Haenszel method. For continuous outcomes, mean differences (MD) with 95% confidence intervals (CI) were calculated using the inverse variance method. A random effects model was used for all analyses. Statistical analysis was performed using R version 4.1.1 (R Foundation for Statistical Computing, Vienna, Austria) and the meta-package, with results visualised in forest plots. Statistical significance was defined as a p-value ≤ 0.05. Heterogeneity was assessed using the I² statistic and visual inspection of forest plots.

### Assessment of risk of bias and certainty of evidence

Risk of bias assessment was independently performed by the two reviewers by applying the ROBINS-I tool for non-randomized studies and the RoB-2 tool for randomized trials [17]. Both reviewers then evaluated the certainty of evidence using GRADEpro Software (McMaster University and Evidence Prime Inc., Ontario, Canada [18]. Disagreements were resolved through consensus.

## Results

### Study characteristics

A total of 242 articles were identified through the systematic search. Following title and abstract screening, 26 full-text articles were reviewed for eligibility. Ultimately, four studies met the inclusion criteria and were incorporated into this meta-analysis [19–22]. A detailed overview of the study selection process is provided in the PRISMA flow diagram (Fig. 1). Of the included studies, one was an RCT, while the remaining three were retrospective observational cohort studies, each utilizing propensity score matching (PSM) to mitigate baseline imbalances. For the present meta-analysis, exclusively the matched datasets were utilized for all included studies, even in cases where unmatched results were also published.

### Patient characteristic

**Table 1:** Patient characteristics. Age, BMI, preoperative haemoglobin, and creatinine/gfr are reported as mean ± standard deviation (SD) for all studies except for Zaid et al., where results are presented as median with interquartile range (IQR)

Study	Number of patients (TXA/Control)	Age (TXA/Control)	Male sex (TXA/Control)	BMI (TXA/Control)	>T2 disease (TXA/Control)	Neoadjuvant chemotherapy (TXA/Control)	Preoperative Hemoglobin (TXA/Control)	Preoperative Creatinine/GFR (TXA/Control)
Zaid et al. (2016)	103 / 200	69 (61–75) / 69 (61–76)	88% / 81%	27 (25–31) / 28 (25–32)	28%/29%	18% / 18%	13.5 (12.5–14.6) / 13.6 (12.7–14.6) g/dL	Not reported
Ahmed et al. (2024)	468 / 468 (matched)	69 (62–73) / 65 (59–71)	Not reported	Not reported	22%/22%	30% / 30%	Not reported, but matching criterion	Not reported
Breau et al. (2024)	178 / 175	70 (64–75) / 68 (62–76)	74% / 75%	27 (25–30) / 28 (25–32)	19%/15%	39% / 44%	131 (116–144) / 129 (115–143) g/L	87.5 (74–107.5) / 88 (71–109) µmol/L
Egen et al. (2024)	32 / 32 (matched)	68 (10) / 72 (9)	84% / 66%	28 (5) / 27 (5)	34%/53%	34% / 38%	13.2 (2.1) / 13.0 (2.1) g/dL	1.1 (0.3) / 1.0 (0.4) mg/dL

### Overall and intraoperative transfusions

**Fig. 2:**
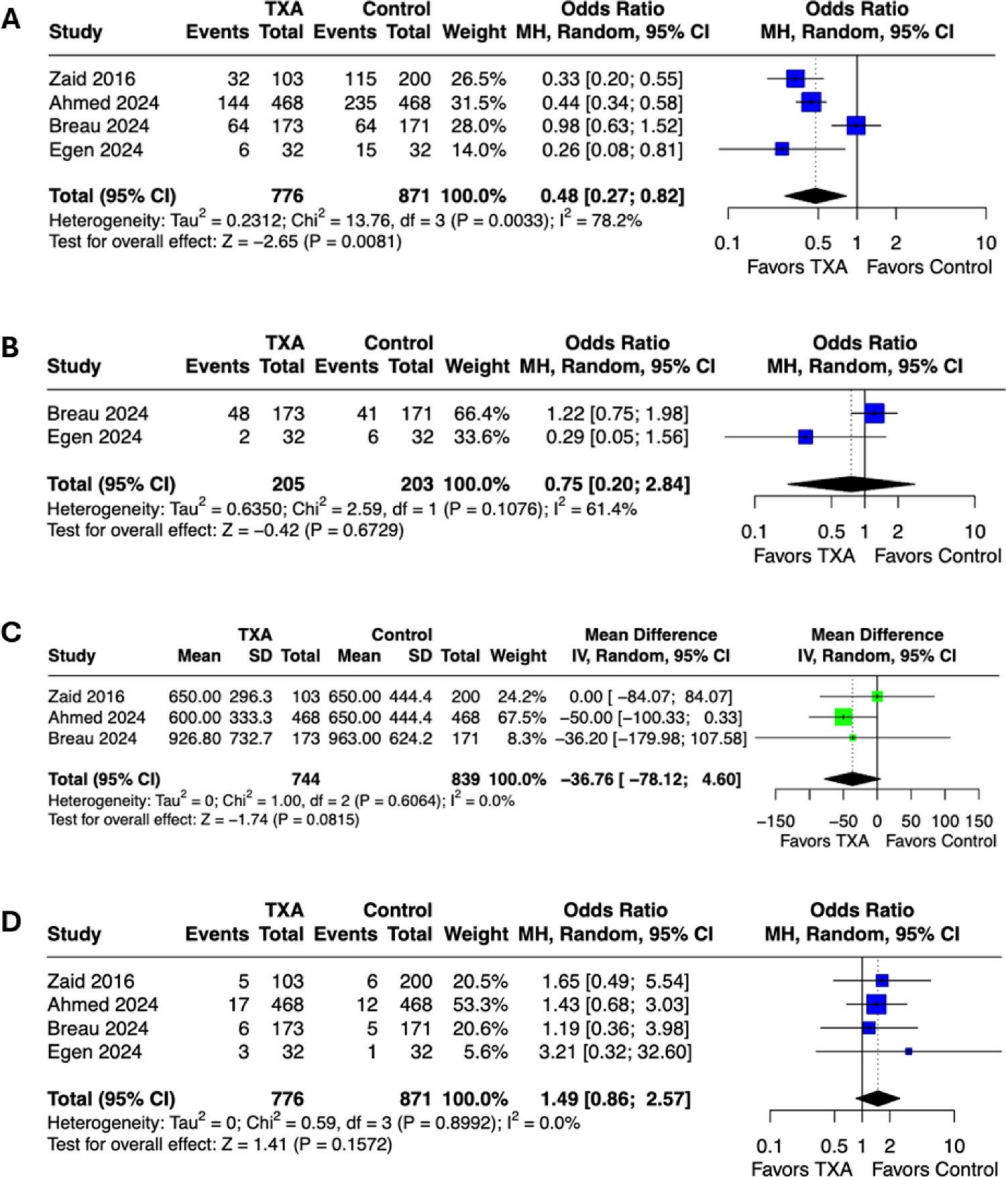
Forrest plots of perioperative outcomes in the TXA group compared to control. A -Overall transfusion rate. B -intraoperative transfusions. C -estimated blood loss. D -venous thromboembolism. (TXA -tranexamic acid, CI -confidence interval)

Of the four studies included in this meta-analysis, only two explicitly reported the timing of blood transfusions (BT) [19, 20]. To ensure clarity, we pooled all reported transfusions (intraoperative and up to 30 days postoperative) across all studies for the primary analysis of overall PBT (Fig. 2). The combined analysis demonstrated a significantly lower overall PBT rate in the TXA group compared to controls (OR 0.48, 95% CI 0.27–0.82; *p* = 0.008). The heterogeneity between the included studies was (Q = 13.76, df = 3, *p* = 0.003, I² = 78%). A separate analysis focusing specifically on intraoperative BT found no significant difference between the TXA and control groups (OR 0.75, 95% CI 0.20–2.84; *p* = 0.67; Fig. 2). Heterogeneity between studies was moderate with an I² value of 61% and a p-value of 0.11.

### Blood loss

Although the difference did not reach statistical significance, estimated blood loss (EBL) was numerically lower in the TXA group compared with controls (MD -37 mL; 95% CI -78 to 5; *p* = 0.08; Fig. 2). Heterogeneity among the included trials was minimal (Q = 1.00, df = 2, *p* = 0.61, I² = 0%), indicating consistent results across trials.

### Adverse events

The rate of VTE did not differ significantly between the TXA and control groups (OR 1.49, 95% CI 0.86–2.57; *p* = 0.16; Fig. 2). No heterogeneity was observed among the included studies (I² = 0%, Q = *0.59 df.=3*, *p* = 0.90). Two studies further differentiated between the types of thrombotic events [19, 21]; similarly, no significant differences were found in the incidence of deep venous thrombosis or pulmonary embolism between the TXA and control groups (OR 1.56, 95% CI 0.67–3.65; *p* = 0.30, and OR 1.50, 95% CI 0.61–3.70; *p* = 0.38, respectively; see supplementary Fig. 1). No heterogeneity was observed for either outcome (Q = 0.25, df = 1, *p* = 0.61, I² = 0% and Q = 0.04, df = 1, *p* = 0.84, I² = 0% respectively; see supplementary material).

### Oncological outcomes

Oncological outcomes were reported in only one study, which precluded a pooled analysis. However, given the notable findings, we have chosen to highlight them here: Ahmed et al. reported 3-year overall survival rates of 76.1% (95% CI, 71.8–80.7%) in the TXA group versus 65.9% (95% CI, 61.7–70.5%) in the control group, and 3-year cancer-specific survival rates of 82.5% (95% CI, 78.6–86.6%) versus 73.4% (95% CI, 69.3–77.7%) respectively [21].

### Risk of bias and certainty of evidence

**Fig. 3:**
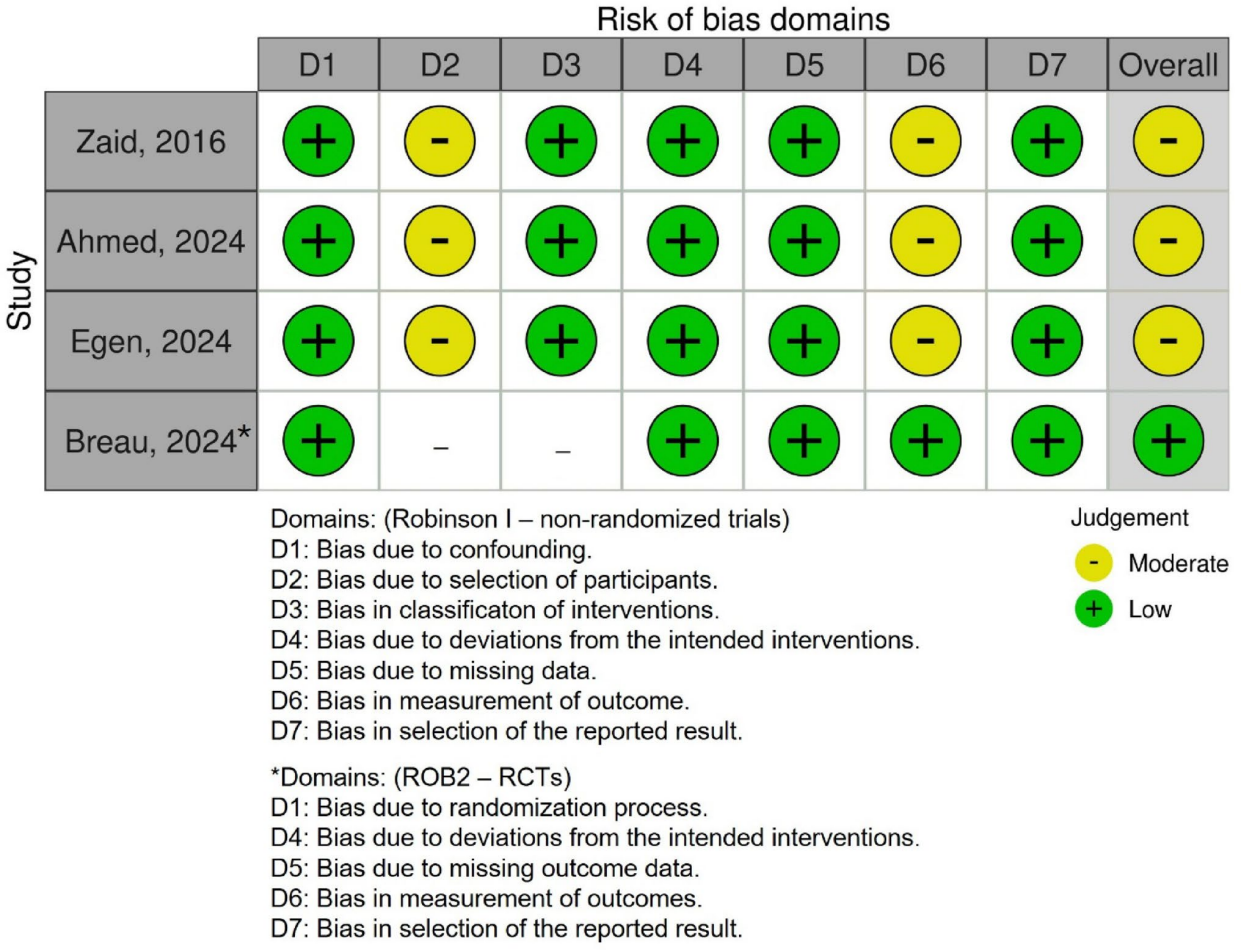
Risk of Bias assessment for the included studies

According to the GRADE assessment, the certainty of evidence was moderate, mainly due to serious indirectness stemming from variations in the TXA dosing regimen across studies (Table 1). Additionally, the retrospective study design of three of the four included studies further lowered the quality of evidence. However, given that only four studies were available, the overall strength of cumulative evidence according to GRADE must be interpreted with caution (Table 2). The risk of bias assessment is illustrated in a traffic light plot (Fig. 3).

**Table 2:**
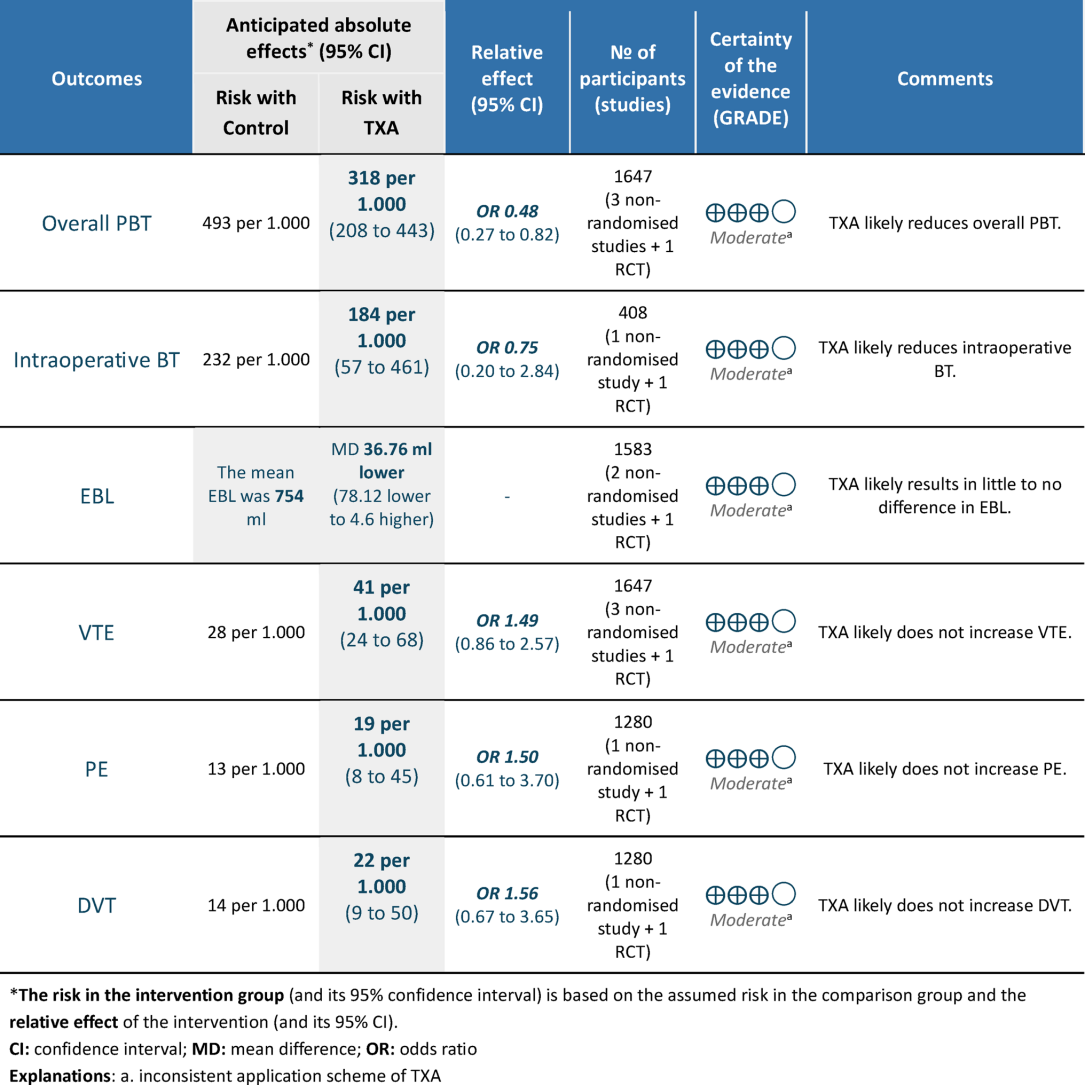
Summary of Findings Table and Quality of Evidence Assessment

PBT -perioperative blood transfusion, BT -blood transfusion, EBL -estimated blood loss, VTE -venous thromboembolism, PE -pulmonary embolism, DVT -deep vein thrombosis, CI -confidence interval, TXA -tranexamic acid, OR -odds ratio, RCT -randomised controlled trial

## Discussion

This systematic review and meta-analysis evaluated the impact of TXA administration on intra- and postoperative outcomes in patients undergoing RC. Our results show that overall TXA is associated with a significant reduction in the need for PBT, with no conclusive evidence of an increased risk of VTE. These results are consistent with the growing body of evidence supporting the utility of TXA in a variety of surgical settings and, for the first time, provide robust data specifically supporting its use in RC. However, it is important to note that the only RCT included in this analysis did not demonstrate a significant reduction in PBT requirements with TXA, whereas the retrospective studies suggested a benefit. This discrepancy highlights the limitations of relying on lower-quality evidence and emphasises the risk of overestimating treatment effects in observational data. Therefore, while our pooled analysis suggests that TXA may reduce the need for PBT, this conclusion must be interpreted with caution: currently, the highest-quality evidence available does not confirm this benefit, emphasising the urgent need for additional high-quality RCTs. In addition, the substantial heterogeneity across trials for the PBT endpoint - and moreover the contrast with results from other surgical specialties where transfusion rates have been reduced by up to 40% [9] - may be partly due to the routine use of perioperative anticoagulation in patients undergoing RC. Available information on anticoagulation protocols varied considerably across studies. Zaid et al. did not report standardised postoperative regimens [22]. Ahmed et al. noted that all patients received 28 days of prophylaxis, primarily with enoxaparin and less frequently with apixaban; however, dosing details were not provided [21]. Breau et al. reported that 84% of patients received intraoperative subcutaneous heparin, but no information on postoperative anticoagulation was provided [19]. Egen et al. followed Enhanced Recovery After Surgery (ERAS) recommendations, initiating heparin prophylaxis on the evening of surgery and continuing it for four weeks; however, the type and dosing were not specified [20]. It is known that the type of anticoagulation alone can influence bleeding risk and transfusion requirements [23]. In addition, it is also possible that perioperative anticoagulation attenuates the haemostatic effects of TXA; however, clinical evidence for this interaction, especially in RC patients, is lacking. In addition to differences in perioperative anticoagulation protocols, other factors may have contributed to the substantial heterogeneity (I² = 78%) observed in the PBT outcome. Firstly, variation in surgical approach is a likely source of inconsistency. Most studies exclusively investigated open RC, whereas the study by Ahmed et al. included a proportion of robotic-assisted cases (20.1% of TXA recipients vs. 8.1% of controls) [21]. The known lower transfusion rates associated with robot-assisted RC may partly explain the heterogeneity observed between studies [24, 25]. Secondly, differences in institutional transfusion triggers could have contributed to heterogeneity. While Breau et al. stated that transfusion decisions were at the discretion of blinded surgical teams, Zaid et al. cited non-standardised transfusion criteria as a limitation of their study [19, 22]. This suggests that variability in transfusion practices between centres may have affected the outcomes. Thirdly, baseline haematological status varied between studies. Zaid et al. reported that nearly half of TXA recipients (51/103) were anaemic, with a haemoglobin level below 13.5 g/dL at baseline [22]. Breau et al. reported that 51 out of 173 patients had a preoperative haemoglobin value below 12.0 g/dL [19]. Ahmed and Egen reported means/medians of preoperative haemoglobin but did not specify anaemia thresholds [20, 21]. Taken together, these data suggest that variability in surgical technique, institutional transfusion practices, and baseline anaemia prevalence likely contributed to the observed heterogeneity, which complicates the interpretation of pooled PBT outcomes. Despite the heterogeneity of the data, it is important to emphasise that even modest reductions in transfusion rates can have significant clinical and economic benefits, given the high risk of bleeding during RC and the risks and costs of PBT as outlined above. Beyond the increased risk of intraoperative bleeding, RC patients also face an intrinsically increased risk of VTE due to cancer-related prothrombotic mechanisms and extensive surgical trauma during RC [26–28]. This underscores the need to balance the haemostatic benefits of TXA against its prothrombotic risks in this vulnerable cohort. The current meta-analysis did not detect a statistically significant increase in thromboembolic complications, consistent with prior large-scale meta-analyses across various surgical disciplines [29–32]. These findings suggest that TXA, when used alongside standard thromboprophylaxis, does not appear to amplify baseline thrombotic risk [33].

It must be noted that the TXA dosing regimen differed between the included studies (Supplementary Table 1). Three studies [19, 21, 22] administered TXA as an intravenous bolus of 10 mg/kg at the start of surgery, followed by a continuous infusion. However, the maintenance dose differed with Breau et al. using 5 mg/kg/h, while Ahmed et al. and Zaid et al. applied 2 mg/kg/h. In contrast, Egen et al. employed a simpler regimen consisting of a single 1 g intravenous bolus preoperatively, without a continuous infusion. In a large-scale meta-analysis focusing on the application of TXA in urologic surgery Kim et al. drew attention to this fact [10]. In their analysis intravenous administration of TXA at doses of 1 g or more was the most frequently used approach, applied in 14 out of the 26 studies. To account for variability in dosing protocols, a sensitivity analysis was performed, excluding studies that employed alternative regimens. This adjustment did not significantly alter the association between TXA use and outcomes: EBL was still markedly reduced (MD -131.5 ml; 95% CI: -211.6 to -51.40; *p* < 0.001), as was the need for PBT (RR = 0.34; 95% CI: 0.21–0.56; *p* < 0.035). Thus, the dosing regimen may not significantly affect the efficacy of TXA. A balance needs to be sought between clinical feasibility and maintaining an appropriate dose-response relationship. High-quality evidence for a standardized dosing and administration protocol is still lacking.

This analysis was unable to draw conclusions about the oncological effects of TXA, as survival outcomes have only been reported in one single retrospective study [21]. While this study suggested an apparent improvement in overall and cancer-specific survival, with 3-year OS rates of 76% in TXA recipients versus 66% in nonrecipients, and CSS rates of 83% versus 73% (*P* < 0.01), these associations were no longer statistically significant after multivariable adjustment for key confounders, including Charlson comorbidity index, adjuvant chemotherapy, variant histology and PBT. Nevertheless, it is important to acknowledge the underlying biological rationale linking transfusion rates with oncological outcomes. While in other surgical contexts TXA has been proposed as a direct anticancer agent [34, 35], in the present setting a more plausible rationale is its indirect effect through the reduction of perioperative transfusion requirements. In this way, TXA may mitigate transfusion-related immunosuppression, which has been linked to adverse oncological outcomes. However, in the context of the present meta-analysis, this remains a hypothetical consideration that requires confirmation in prospective studies.

This meta-analysis is not free of limitations. Most of the included studies were retrospective observational cohort studies, often relying on historical controls, which increases the risk of selection bias and data inaccuracy. Although one RCT provided high quality evidence, its findings contrasted with those of the retrospective studies regarding some endpoints, highlighting the need for additional RCTs. Furthermore, variability in TXA dosing, as outlined above, complicated direct comparisons and underscores the need for standardized, well-documented regimens to carry out further comparable studies. Finally, while most available data focus on open RC, it remains unclear whether similar benefits and safety profiles would extend to minimally invasive approaches such as robotic-assisted RC. As pointed out before, only one of the trials included robotic-assisted RCs [21], a detailed analysis of this subgroup was not feasible. However, existing RCTs comparing robotic-assisted and open RC provide valuable insight into perioperative differences. In the RAZOR trial, robotic-assisted RC was associated with significantly lower median blood loss (300 mL vs. 700 mL, *p* < 0.0001) and lower perioperative transfusion rates (24% vs. 45%, *p* = 0.0002) [24]. Similarly, the iROC trial reported a median intraoperative blood loss of 200 mL (IQR 125–375) with robotic RC compared to 550 mL (IQR 400–859) with open RC, along with lower postoperative transfusion rates (7% vs. 12%) [25]. These findings suggest that robotic-assisted RC may offer perioperative advantages in terms of PBT requirements. Given the increasing use of robotic surgery, future studies should evaluate the generalisability of our findings and the role of TXA across different surgical platforms.

## Conclusion

In conclusion, this meta-analysis supports the role of TXA as a potential strategy to reduce the need for PBT in patients undergoing RC, without clear evidence of increased thromboembolic risk. These findings support the considerate incorporation of TXA into perioperative management. Future research should ideally consist of large, multicentre RCTs with adequate representation of robotic-assisted RC and with standardized TXA dosing and anticoagulation protocols, specifically designed to confirm TXA´s efficacy in minimizing PBT requirements and to assess long-term safety as well as potential oncological outcomes.

## Supplementary Information

Below is the link to the electronic supplementary material.

Supplementary Material 1

Supplementary Material 2

## Data Availability

No datasets were generated or analysed during the current study.
