# Supplementary material for: Tranexamic acid in radical cystectomy: a systematic review and meta-analysis of efficacy and safety
Source: World J Urol. 2025 Sep 18;43(1):560. doi: 10.1007/s00345-025-05939-0 (PMC12446414; doi:10.1007/s00345-025-05939-0)
Supplement: Supplementary file 1 — Supplementary Material 1 [file 345_2025_5939_MOESM1_ESM.pdf]

Systematic literature search

Search report

Purchaser
Miss Dr. Maurin Mangold

Assigned 12.12.2024

Delivered 30.01.2025

Executed by
Maurizio Grilli M.A.L.I.S.
Library for the Medical Faculty of Mannheim, University of Heidelberg
Universitätsmedizin Mannheim
Theodor-Kutzer-Ufer 1-3
Haus 42
maurizio.grilli@medma.uni-heidelberg.de
tel. 0621/383-3720

Topic

Time needed

Date	Position	Hours
09.01.25	Search draft	0,3
21.01.25	Clarification of open questions during an interview	0,3
30.01.25	Completion of the literature research	1,1
		**Total**
		2,1

Main concepts

P

	Cystektomie	

I

	Tranexamsäure	

No Limits:

Citation searching
No citation searching
No additional studies were sought
No other literature reviews was reused

Search guide overview

1	P	
2	I	
3	#1 AND #2	

Databases and platforms involved

- PubMed (via NCBI)
- EMBASE (via Elsevier)
- Cochrane Library (via Wiley)
- Web of Science (via Clarivate)
- ClinicalTrials.Gov (via www.clinicaltrials.gov)
- ICTRP (via https://trialsearch.who.int/Default.aspx)
- MedRxiv BioRxiv (über Europe PMC)

Results report
The results were saved and deduplicated in Endnote. For this, the settings DOI/ Author, Year, Title, Secondary Title/ Author, Year, Title, Pages/ Title/ Author, Year were applied. However, some items may appear more than once.

The hits are sorted by database in Endnote. The PubMed hits were the first to be exported to Endnote. These are therefore preferred for deduplication. This means that in the case of duplicates, entries from other databases are removed first. The hits in the trash folder are the removed duplicates.

The hit count for each database in this report relates to the status before deduplication in EndNote.

PubMed

Hits	Date
11	30.01.2025

P

1	**"Cystectomy"[Mesh] OR**<br>Cystectom*[tiab] OR<br>Cystoprostatectom*[tiab] OR<br>"cysto prostatectom*"[tiab] OR<br>RCP[tiab]	26052

I

2	**"Tranexamic Acid"[Mesh] OR**<br>tranexam*[tiab] OR<br>TXA[tiab]	9440

Strings
1-2 as in the tables above

Field	String	Hits
3	#1 AND #2	11

Embase

Hits	Date
13	30.01.2025

P

1	**'cystectomy'/exp OR**<br>Cystectom*:ti,ab,kw OR<br>Cystoprostatectom*:ti,ab,kw OR<br>"cysto prostatectom*":ti,ab,kw OR<br>RCP:ti,ab,kw	51634

I

2	**'tranexamic acid'/exp OR**<br>tranexam*:ti,ab,kw OR<br>TXA:ti,ab,kw	27031

Strings

1-2 as in the tables above

Field	String	Hits
3	#1 AND #2	61

Embase filter to switch off PubMed

4	#3 NOT ([medline]/lim OR [pubmed-not-medline]/lim)	29

Embase filter to exclude document types not of interest

5	#4 NOT ('Conference Abstract'/it OR 'Note'/it OR 'chapter'/it)	13

Cochrane Library

Hits	Date
Reviews: 0<br>Trials: 8	30.01.2025

P

1	**[mh "Cystectomy"] OR**<br>Cystectom*:ti,ab,kw OR<br>Cystoprostatectom*:ti,ab,kw OR<br>cysto prostatectom*:ti,ab,kw OR<br>RCP:ti,ab,kw	2523

I

2	**[mh "Tranexamic Acid"] OR**<br>tranexam*:ti,ab,kw OR<br>TXA:ti,ab,kw	4562

Strings

1-2 as in the tables above

Field	String	Hits
3	#1 AND #2	8

Web of Science Core Collection

Hits	Date
9	30.01.2025

P

1	Cystectom* OR<br>Cystoprostatectom* OR<br>"cysto prostatectom*" OR<br>RCP	

I

2	tranexam* OR<br>TXA	

Strings

Field	String	Hits
1	ALL=(Cystectom* OR Cystoprostatectom* OR "cysto prostatectom*" OR RCP)	35979
2	ALL=(tranexam* OR TXA)	11509

3	#1 AND #2	9

Publication year from 2013

5	Publication year from 01-01-2013 to 12-31-2037	

ClinicalTrial.gov
http://www.clinicaltrials.gov/

Hits	Date
0	30.01.2025

P

1	Cystectomy OR<br>Cystoprostatectomy OR<br>"cysto prostatectomy" OR<br>RCP	

I

2	tranexam OR<br>TXA	

Strings

Field	String	Hits
1	(<br>Cystectomy OR Cystoprostatectomy OR "cysto prostatectomy" OR RCP<br>)	768
2	AND<br>(<br>Other terms: tranexam OR TXA<br>)	379

3	1 AND 2	0

International Clinical Trials Registry Platform ICTRP (WHO Trials)
https://trialsearch.who.int/ (simple)
https://trialsearch.who.int/AdvSearch.aspx (advanced)

**Hits**	**Date**
advanced mode: 0	30.01.2025

P

1	Cystectomy OR<br>Cystoprostatectomy OR<br>"cysto prostatectomy" OR<br>RCP	

I

2	tranexam OR<br>TXA	

Strings (in advanced mode)

**Fields**	**String**	**Hits**
1 (Title)	tranexam OR TXA	112
2 (Condition)		
3 (Intervention)	Cystectomy OR Cystoprostatectomy OR "cysto prostatectomy" OR RCP	244

**Fields**	**String**	**Hits**
1 (Title)		
2 (Condition)	tranexam OR TXA	9
3 (Intervention)	Cystectomy OR Cystoprostatectomy OR "cysto prostatectomy" OR RCP	244

4	1 AND 3	0
5	2 AND 3	0

MedRxiv BioRxiv (über Europe PMC)
https://europepmc.org/

Hits	Date
198	30.01.2025

P

1	Cystectom* OR<br>Cystoprostatectom* OR<br>"cysto prostatectom*" OR<br>RCP	

I

2	tranexam* OR<br>TXA	

Strings

Field	String	Hits
	(<br>Cystectom* OR Cystoprostatectom* OR "cysto prostatectom*" OR RCP<br>)	57962
	AND<br>(<br>tranexam* OR TXA<br>)	21941
	AND<br>(PUBLISHER:MedRxiv OR PUBLISHER:BioRxiv)	198
