# Supplementary material for: Tranexamic acid in radical cystectomy: a systematic review and meta-analysis of efficacy and safety
Source: World J Urol. 2025 Sep 18;43(1):560. doi: 10.1007/s00345-025-05939-0 (PMC12446414; doi:10.1007/s00345-025-05939-0)
Supplement: Supplementary file 2 — Supplementary Material 2 [file 345_2025_5939_MOESM2_ESM.docx]

# Supplementary material

**Supplementary Figure 1:** Forest plots of perioperative outcomes in the TXA group compared to control. A -Pulmonary embolism. B -Deep venous thrombosis.

| **Study** | **Bolus** | **Maintenance infusion** |
| --- | --- | --- |
| Zaid et al. (2016) | 10 mg/kg IV | 2 mg/kg/h during surgery,  adjusted for renal impairment (Cr <1.6 mg/dL) |
| Ahmed et al. (2024) | 10 mg/kg IV | 2 mg/kg/h during surgery,  adjusted for renal impairment (Cr <1.6 mg/dL) |
| Breau et al. (2024) | 10 mg/kg IV within 10 min before incision | 5 mg/kg/h until skin closure (±20 min),  placebo received matched Saline infusion |
| Egen et al. (2024) | 1 g IV 30 min before surgery | single bolus regimen, no maintenance infusion |

**Supplementary Table 1:** Tranexamic acid (TXA) dosing regimens across included studies. Cr = Creatinine.

| **First Author (Year)** | **Title (abbreviated)** | **Journal/ Source** | **Reason for Exclusion** |
| --- | --- | --- | --- |
| Ishii (2020) | TXA during Pancreaticoduodenectomy | *BMJ Open* | Non-cystectomy cohort |
| Sentilhes (2020) | TRAAP2 -TXA for cesarean delivery | *BMC Pregnancy Childbirth* | Non-cystectomy cohort |
| Sentilhes (2015) | TRAAP -TXA for vaginal delivery | *BMC Pregnancy Childbirth* | Non-cystectomy cohort |
| Prasad (2018) | TXA in abdominal oncosurgery | *J Anaesthesiol Clin Pharmacol* | Non-cystectomy cohort |
| Maibom (2023) | BORARC trial (robotic vs. open RC) | *Pilot Feasibility Stud* | No TXA intervention |
| Wright (2018) | TXA in major oncologic surgery | *Ann Surg Oncol* | Non-cystectomy cohort |
| Abel (2014) | Perioperative transfusion in RC | *Eur Urol* | No TXA intervention |
| Eden (2021) | Consensus on hemostatic powders | *Updates Surg* | No TXA intervention/ non-cystectomy cohort |
| Kim (2023) | TXA in urological surgeries (SR/MA) | *Can Urol Assoc J* | Non-cystectomy cohort |
| Moschini (2017) | Blood transfusion & survival in RC | *Transl Androl Urol* | Abstract only |
| Yang (2024) | Hyperbaric oxygen for hemorrhagic cystitis | *J Clin Med* | Non-cystectomy cohort |
| Akkaranurakkul (2021) | TXA in laparoscopic cystectomy for endometriosis | *Pilot Feasibility Stud* | Non-cystectomy cohort |
| Bibi (2023) | TXA in radical cystoprostatectomy | *Eur Urol* | Abstract only or study in progress |
| IRCT Trial (2023) | Fibrinogen vs. TXA in RC (abstract) | *WHO Trial Registry* | Abstract only or study in progress |
| Punjani (2013) | Blood transfusion & hemostatic agents in RC | *Can Urol Assoc J* | No TXA intervention (only survey on TXA use) |
| Cleveland (2023) | TXA for percutaneous nephrolithotomy | *Cochrane Database Syst Rev* | Non-cystectomy cohort |
| Koh (2021) | TXA in extrahepatic abdominal surgery (MA) | *BJS Open* | Non-cystectomy cohort |
| Jendoubi (2017) | TXA in TUR procedures | *Prog Urol* | Non-cystectomy cohort |
| Abu-Zaid (2022) | TXA in hysterectomy (SR/MA) | *Obstet Gynecol Sci* | Non-cystectomy cohort |
| Adv Biomed (2022) | TXA in hysterectomy (TA/TV) | *Adv Biomed Res* | Non-cystectomy cohort |
| HemaSphere (2022) | Abstract Book, 27th EHA Congress | *HemaSphere* | Abstract only |
| Balik (2022) | RARPEX -TXA in robotic prostatectomy | *Study protocol* | Non-cystectomy cohort |

**Supplementary Table 2:** Reasons for exclusion after full-text research.
